# Draft Genome Assembly of *Acinetobacter baumannii* ATCC 19606

**DOI:** 10.1128/genomeA.00832-14

**Published:** 2014-08-21

**Authors:** Karen W. Davenport, Hajnalka E. Daligault, Timothy D. Minogue, David C. Bruce, Patrick S. G. Chain, Susan R. Coyne, James G. Jaissle, Galina I. Koroleva, Jason T. Ladner, Po-E Li, Gustavo F. Palacios, Matthew B. Scholz, Hazuki Teshima, Shannon L. Johnson

**Affiliations:** a Los Alamos National Laboratory, Los Alamos, New Mexico, USA; b United States Army Medical Research Institute of Infectious Diseases—Diagnostic Service Division (USAMRIID-DSD), Fort Detrick, Maryland, USA; c United States Army Medical Research Institute of Infectious Diseases—Center for Genome Sciences (USAMRIID-CGS), Fort Detrick, Maryland, USA

## Abstract

*Acinetobacter baumannii* is an emerging nosocomial pathogen, and therefore high-quality genome assemblies for this organism are needed to aid in detection, diagnostic, and treatment technologies. Here we present the improved draft assembly of *A. baumannii* ATCC 19606 in two scaffolds. This 3,953,621-bp genome contains 3,750 coding regions and has a 39.1% G+C content.

## GENOME ANNOUNCEMENT

A common nosocomial pathogen with high morbidity among military personnel involved in combat, *Acinetobacter* has generated a great deal of interest (1, 2). Species of this genus are known to be multiply antibiotic resistant and cause various human tissue infections (e.g., respiratory and urinary tract infections and bacteremia) (3, 4). We sequenced and assembled the *Acinetobacter baumannii* type strain into two scaffolds (chromosome in 17 contigs, plasmid in 1 contig).

High-quality genomic DNA was extracted from purified isolates of each strain using QIAgen Genome Tip-500 at USAMRIID-DSD. Specifically, 100-ml bacterial cultures were grown to stationary phase and nucleic acid was extracted per the manufacturer’s recommendations. Sequence data generated for the draft genome included a combination of Illumina and 454 technologies (5, 6). For this genome assembly, we constructed and sequenced an Illumina library of 100-bp reads to high coverage (295-fold genome-coverage) and a separate long-insert paired-end library (average insert size 12,913.6 ± 3,228.4 bp, run on a Roche 454 Titanium platform to 18-fold genome coverage). The two libraries were assembled together in Newbler (Roche), and the consensus sequences were computationally shredded into 2-kbp overlapping fake reads (shreds). The raw reads were also assembled in Velvet, and those consensus sequences were computationally shredded into 1.5-kbp overlapping shreds (7). Draft genome data from all platforms were then assembled together with Allpaths, and the consensus sequences were computationally shredded into 10-kbp overlapping shreds (8). We then integrated the Newbler consensus shreds, Velvet consensus shreds, Allpaths consensus shreds, and a subset of the long-insert read pairs using parallel Phrap (High Performance Software, LLC). Possible misassemblies were corrected and some gap closure accomplished with manual editing in Consed (9–11).

Automatic annotation for each genome utilized an Ergatis-based workflow at LANL with minor manual curation. Annotation located 3,750 coding genes, 61 tRNA,s and 7 rRNAs. The final 3,953,621-bp assembly has 39.1% G+C content and 1 expected plasmid (16,340-bp).

### Nucleotide sequence accession number.

The final sequence has been deposited to GenBank under the accession number JMRY00000000.
